# Evolutionary origins of spinal cord tumors: A cross-species systematic review

**DOI:** 10.1093/emph/eoaf028

**Published:** 2025-10-14

**Authors:** Gabriel Urreola, Alan Harris, Michael Le, Jose Castillo, Dharminder Ojla, Allan R Martin, Kee D Kim, Richard L Price

**Affiliations:** School of Medicine, University of California, Davis, CA, USA; University of Louisville, School of Medicine, Louisville, KY, USA; School of Medicine, University of California, Davis, CA, USA; Department of Neurological Surgery, University of California, Davis, CA, USA; School of Medicine, University of California, Davis, CA, USA; Department of Neurological Surgery, University of California, Davis, CA, USA; Department of Neurological Surgery, University of California, Davis, CA, USA; Department of Neurological Surgery, University of California, Davis, CA, USA

**Keywords:** glial tumors, cancer, evolution, spinal cord, spinal cord tumors

## Abstract

**Objective:**

Intradural spinal cord tumors, particularly intramedullary glial neoplasms such as astrocytomas, ependymomas, and oligodendrogliomas, are rare in humans but pose significant diagnostic and therapeutic challenges. Despite their clinical importance, little is known about their evolutionary origins or cross-species presentation. This study aimed to synthesize existing veterinary literature on spontaneous intradural spinal tumors in non-human animals to uncover patterns in clinical symptoms, tumor distribution, and histopathological features that may inform human spinal oncology.

**Methods:**

We conducted a systematic review of Medline, Embase, and Web of Science using a Preferred Reporting Items for Systematic Reviews and Meta-Analyses-guided approach. Studies were screened using Covidence by multiple independent reviewers. Inclusion criteria required spontaneous, histologically confirmed intradural spinal cord tumors in non-human species. Data extracted included species, tumor type, anatomical location, presenting symptoms, and histopathology. Phylogenetic and life history data were incorporated to explore evolutionary trends.

**Results:**

A total of 109 studies describing 155 cases across 11 animal species were included. Astrocytomas (*n* = 37), oligodendrogliomas (*n* = 31), and ependymomas (*n* = 24) were the most common tumor types, with a notable bias toward the cervical spinal cord. Neurological symptoms were consistent with human intradural tumors: 42% of cases presented with limb paralysis and 28% with ataxia. The most recent common ancestor among affected species was the amniote, suggesting an evolutionary origin of spinal glial vulnerability dating back over 340 million years.

**Conclusions:**

This is the first cross-species synthesis of spontaneous intradural spinal cord tumors in non-human animals. The evolutionary conservation of tumor types and symptom patterns highlights opportunities to develop genetically informed animal models and identify early diagnostic markers. Expanding comparative tumor databases may enhance our understanding of spinal cord oncogenesis and support novel therapeutic strategies in both veterinary and human medicine.

## INTRODUCTION

Cancers concerning the spinal cord exhibit a wide range of presentations, often leading to diagnostic delays. Many intradural spinal cord tumors are not symptomatic until they exhibit significant mass effect, which can obscure early detection [1, 2]. Additionally, spinal tumor symptomatology can be non-specific, further delaying diagnosis [3, 4]. Other factors contributing to this difficulty include a variety of susceptible cell types, ranging from Schwann cells and ependymal cells to glial cells and osteoblasts that all have different properties and malignancy potential [5, 6]. The spinal cord spans from the cervicomedullary junction at the base of the skull to the conus medullaris and cauda equina within the lumbosacral spinal canal and is responsible for relaying both autonomic and somatic innervation throughout the body [5]. This critical function creates a unique challenge in identifying intradural tumors, as early symptoms often resemble more common conditions and may not immediately raise clinical suspicion [2].

In humans, survival rates for spinal tumors vary significantly based on tumor type and malignancy. The 5-year survival rate for intramedullary malignant spinal cord tumors ranges from ~ 71% to 86%, while benign tumors are associated with much higher survival rates [5]. Despite this, delayed diagnosis remains a significant clinical concern. Intradural spinal tumors are typically small due to anatomical constraints within the spinal canal, but their location often leads to early neurological symptoms even at modest sizes [7]. In contrast, metastatic or extradural tumors may grow larger before detection, sometimes mimicking or surpassing the clinical burden of tumors in more commonly screened sites such as the lung, breast, or prostate [8]. This suggests that both biological behavior and delayed recognition contribute to the morbidity associated with spinal tumors.

Our paper aims to identify signs and symptoms of spinal tumors in animal analogs as a lens to retrospectively apply to human cases. Evolutionary and comparative medicine, in which breakthroughs in one field (e.g. ophthalmology or cardiology) have informed human treatments, can underscore the value of looking beyond humans to uncover mechanisms that may similarly accelerate early detection and treatment of spinal cord tumors.

Given the often-late diagnosis of various spinal tumors, we centered our study on early signs and symptoms of spinal tumors in animal models. A multi-species review allowed us to study degrees of susceptibility to spinal tumors, similarities and differences in histopathology, clinical presentation, tumor marker prevalence, and morbidity. Uncovering congruencies between animal models and humans can help us identify potential avenues for further research and innovation in the treatment and screening of spinal tumors.

## METHODS

### Search strategy & protocol

A search of Medline, Embase, and Web of Science in July of 2024 yielded 1856 potential matches. (i) Web of Science (Topic Search) was queried as: TS = (Ependymoma* OR Ganglioglioma* OR Hemangioblastoma* OR Glioma* OR Glioblastoma* OR Astrocytoma* OR Oligodendroglioma*) AND TS = (Spine* OR ‘Spinal Cord’* OR Intramed* OR Intradur* OR ‘Spinal Cord Neoplas*’ OR ‘Spinal Cord Tumor*’ OR Neuropath*) AND TS = (veterinar* OR ‘veterinary med*’ OR zoo* OR arthropod* OR fish* OR reptile* OR snake* OR amphibian* OR bird* OR avian*). In Embase key terms such as [ependymoma* OR ganglioglioma* OR hemangioblastoma* OR glioma* OR glioblastoma* OR astrocytoma* OR oligodendroglioma* OR ‘ependymoma’/exp OR ‘ganglioglioma’/exp OR ‘hemangioblastoma’/exp OR ‘glioma’/exp OR ‘glioblastoma’/exp OR ‘astrocytoma’/exp OR ‘oligodendroglioma’/exp] [veterinary* OR zoo* OR arthropod* OR fish* OR reptile* OR snake* OR amphibian* OR bird* OR avian* OR ‘veterinary medicine’ OR zoo OR arthropod OR fish OR reptile OR snake OR amphibian OR bird OR avian] [spine* OR spinal* OR cord* OR intramed* OR intradur* OR neuropath* OR spine OR ‘spinal cord’ OR intramedullary OR intradural OR ‘spinal cord tumor’ OR ‘spinal cord disease’ OR ‘neuropathology’] were used. Our search goals were aided by UC Davis School of Medicine librarians to refine our search terms to yield the most accurate returns. The goal was to optimize the number of articles retrieved, encompassing animal cases of spinal tumors while excluding as many human cases as possible. Although the search was intentionally broad and included aquatic taxa, the review was prospectively limited to terrestrial vertebrates. Accordingly, fish records were excluded during screening to preserve comparability across species. Notably, we followed the 2020 Preferred Reporting Items for Systematic Reviews and Meta-Analyses (PRISMA) guidelines and checklist (*Matthew J Page, 2021 PRISMA*) [9].

**Figure 1:**
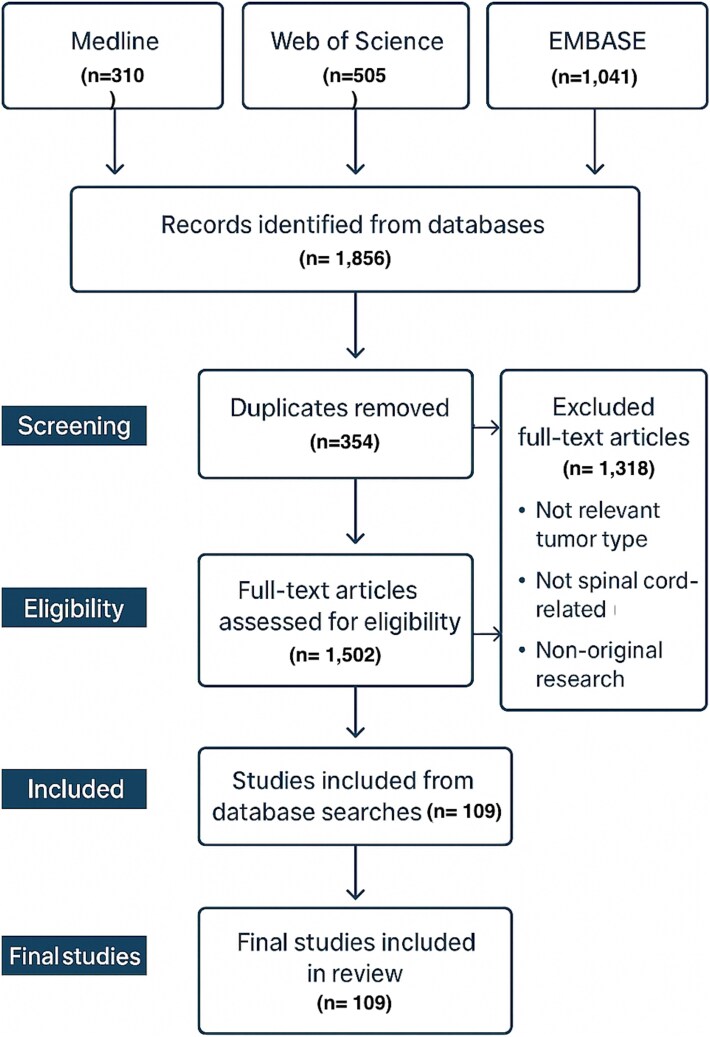
PRISMA flow diagram depicting the article screening and selection process for studies on spontaneous spinal cord tumors in non-human species.

### Inclusion/exclusion criteria

Our initial search yielded 1856 studies and was uploaded to Covidence, a web-based software designed to streamline blinded research article screening and data extraction. We conducted an initial screening with three independent reviewers who screened articles based on title and abstract. Another secondary screening was done by two independent reviewers, in which the full-text article was screened. All disagreements through the two screenings were discussed by two reviewers, and the inclusion/exclusion protocol was reassessed to determine final placement. After both screenings, our final search included 109 articles (Fig. 1). Following the systematic review of articles, a manual search was conducted by reviewing the citations of all articles, and we included any relevant articles in the final search.

Our inclusion criteria specified for histopathologically confirmed intradural spinal cord tumors in non-human species, including both intramedullary and intradural extramedullary lesions, where clearly defined. In keeping with this a priori scope, we restricted the synthesis to terrestrial vertebrates (amniotes); aquatic species (e.g. fish) were excluded because of distinct spinal anatomy/physiology and the paucity of histologically confirmed intradural spinal cord tumor reports. We included case reports, case series, clinical trials, and retrospective studies that had a sufficient breakdown of case information. We excluded any articles that had human cases, or that were not spontaneous (i.e. animal models, xenografts, genetically modified, cell lines). We also excluded any reviews, meta-analyses, conference presentations, abstracts, and non-English articles. Lastly, we excluded any articles that had a speculative diagnosis that was not confirmed by histopathology. Once all articles were finalized, initial demographic data were collected, such as tumor type counts (Table 1) and frequency of specific presenting symptoms (Table 2). Given the nature of our review, we provided a descriptive analysis of our data, emphasizing hypothesis generation. The paucity and inconsistency of animal necropsy data made it difficult to infer epidemiological or causal conclusions. Cross-species modeling of tumor occurrence against life-history variables (e.g. body mass, maximum longevity) was not feasible with the available case-based evidence and was therefore intentionally omitted. In total, there were 155 unique cases across 11 species, identified as spontaneous spinal tumors. The scientific name, common name, age characteristics, sex, lifestyle (i.e. lab, pet, livestock, wild), anatomical location of tumor, neurological symptoms, and molecular/histopathological markers were extracted from each final study.

**Table 1:** Distribution of tumor histologies in 152 spinal cord tumor cases.

**Tumor type**	***n***	**% of total (n = 152)** ^**a**^
Astrocytoma	37	24.3%
Oligodendroglioma	31	20.4%
Ependymoma	24	15.8%
Glioblastoma	19	12.5%
Intradural, NOS^b^	13	8.6%
Schwannoma	8	5.3%
Hemangioblastoma	6	3.9%
Meningioma	4	2.6%
Neuroblastoma	4	2.6%
Polymorphic cell sarcoma	3	1.9%
Lymphoma	2	1.3%
Nephroblastoma	2	1.3%
Choroid plexus tumor	1	0.7%
Adenocarcinoma	1	0.7%

Number and percentage of cases for each histologic subtype, ordered by descending frequency. Percentages are based on the 152 cases with confirmed pathology; cases lacking histologic data are excluded. ‘Intradural, NOS’ indicates tumors without a more specific classification.

a Total excludes cases with ‘n/a’ or missing tumor-type data.

b NOS = not otherwise specified.

**Table 2:** Neurological symptoms in 155 cases.

**Symptom category**	**Specific signs**	***n***	**% of cases**
Motor weakness	Tetraparesis, monoparesis (fore + hind limbs)	28	18.1%
Limb paralysis	Forelimb + hind limb paralysis	33	21.3%
Limb ataxia	Forelimb + hind limb ataxia	40	25.8%
Sensory/reflex deficits	Absent deep tendon reflexes	1	0.6%
Autonomic dysfunction	Anal sphincter paralysis, priapism	2	1.3%
Other	Blindness, lethargy, seizures, n/a	25	16.1%

Number and percentage of cases exhibiting each symptom category. Symptoms are grouped into broad clinical categories for clarity; detailed sign‐level counts are provided in Supplementary Table S1.

**Note:** Detailed breakdown of every specific sign (e.g. ‘hind limb monoparesis n = 4’) is available in Supplementary Table S1.

### Life history information

We leveraged existing databases (AnAge, Animal Diversity Web) for average lifespan, maximum longevity, age of maturation, and body mass for the animals in our dataset. Trait values were extracted for context only; formal comparative analyses were out of scope. Given absent denominators, heterogeneous ascertainment, and inconsistent trait reporting, we did not evaluate tumor frequency as a function of body mass or lifespan.

### Data visualization

We utilized several software to illustrate a comprehensive phylogenetic tree (Phytotree) (Biorender) (iTOL). Our phylogenetic tree illustrates all documented cases of tumors across species and highlights the potential origins and ranges of glial tumor vulnerability.

## RESULTS

Our review included 109 original studies with 155 total cases of spinal cord tumors across 11 species. Most cases were found in dogs and cats (the majority of our dataset consisted of domesticated carnivores). Cases were identified in 10 mammalian species and one reptile species. We documented 14 distinct types of spinal cord tumors, with astrocytomas, oligodendrogliomas, and ependymomas being the most prevalent (37, 31, and 24 cases, respectively). Notably, a few rare spinal tumors were also reported, such as single cases of adenocarcinoma, choroid plexus tumor, and nephroblastoma (see Table 1 for a full list). The cases showed a wide geographical distribution of species across at least 3–4 continents. The most recent common ancestor among all animals in our review was the amniote, suggesting these nervous system tumors have origins dating back an estimated 340 million years.

In Table 2, we summarize the neurological symptoms observed in these animals. The two most frequent symptoms were some forms of ataxia or limb paralysis. Of the 145 total neurological symptoms documented, 42% involved limb paralysis and 28% involved limb ataxia. Other notable presentations included spinal column deformation, generalized weakness, or even a lack of observable neurological symptoms in some cases. As shown in Table 3, the distribution of spinal cord tumors across anatomical regions in the spine was fairly even: 21.48% of tumors occurred in the thoracic region, 22.96% in the cervical region, and 20.74% in the lumbar region. Table 3 also details the specific tumor types observed in each region, allowing for comparison of anatomical prevalence patterns across species. Certain tumor types, such as schwannomas and meningiomas, appeared relatively equally across all spinal regions. On the other hand, our data suggest that tumors such as astrocytomas and oligodendrogliomas may have a bias toward the upper spinal cord, with 54% and 40% of those tumors, respectively, occurring in the cervical region.

**Table 3:** Tumor prevalence by spinal region.

**Spinal region**	***n***	**% of total cases**
Cervical	31	22.9%
Thoracic	29	21.4%
Lumbar	28	20.7%
Full spine	18	13.3%
Thoracolumbar	13	9.6%
Cervicothoracic	8	5.9%
Lumbosacral	4	2.9%
Sacral	3	2.2%
Spinobulbar	1	0.7%

Number and percentage of tumors occurring in each spinal region (*n* = 135 cases with location data). Detailed region‐specific histologic breakdown appears in Supplementary Table S2.

**Note:** For each region, the specific tumor‐type counts are listed in Supplementary Table S2.

## DISCUSSION

In our data, we found that spinal cord tumors are not exclusively a human disease. Spinal cord tumors occur in a wide array of mammals, and we even identified a case in a reptile (snake). The most recent common ancestor among the species in our study was an amniote, a group that includes mammals, birds, and reptiles, suggesting that the vulnerability to spinal cord tumors may have originated over 340 million years ago [10]. This highlights that the oncogenesis of neural tissue has deep evolutionary roots. From an evolutionary perspective, several factors may explain why spinal glial tumors arise across amniote species. Developmental constraints during vertebrate spinal cord formation can create conserved vulnerabilities in glial progenitors. Longevity-related selection pressures, illustrated by Peto’s Paradox, mean that long-lived or large-bodied animals accumulate somatic mutations over extended lifespans, thereby uncovering ancient tumor-suppressor pathways [11]. Finally, the deep conservation of glial proliferation controls and key oncogenes suggests that these molecular circuits, inherited from a common ancestor, continue to predispose diverse lineages to similar neoplasms [12]. Our review demonstrates that neurological symptoms in affected animals occur in patterns like those reported in humans: 42% of animal cases had motor dysfunction, compared to ~ 55% of human patients with intradural tumors presenting with motor deficits (Dang *et al.*, 2012). Tumors in our dataset were found in all major regions of the spine (cervical, thoracic, lumbar) with no strong predisposition to any single region. Among the 14 tumor types identified across species, astrocytomas, oligodendrogliomas, and ependymomas were the most prevalent, totaling 37, 31, and 24 cases, respectively. This predominance of glial-derived tumors across multiple species reinforces their biological relevance and supports the rationale for using animal models to better understand the pathogenesis and progression of these tumors in humans. Although certain spinal cord tumor subtypes, such as intramedullary gliomas, are relatively rare in humans, they are well-characterized in the neurosurgical literature (Dang *et al.*, 2012). Our cross-species data allow for synergy between scientific disciplines; veterinary and zoological findings can supplement human data where human cases are sparse. Any noted parallels with human tumor patterns are qualitative and should be interpreted cautiously; no formal cross-species statistical comparisons with human cohorts were performed.

Notably, different tumor histologies tended to manifest with different clinical patterns across species. Intramedullary glial tumors (like astrocytomas and oligodendrogliomas) often produced severe, diffuse neurological deficits, whereas extramedullary tumors, such as meningiomas or nerve sheath tumors, more commonly caused localized or chronic compression symptoms. Although our sample sizes were limited, these observations suggest that tumor type influences symptomatology and may help clinicians anticipate which symptom patterns indicate a particular tumor. We also found that hind limb dysfunction (paralysis or ataxia) was far more frequently reported than forelimb deficits. This pattern correlated with the anatomical distribution of tumors, as many were located in the thoracolumbar spine. Even cervical tumors often first manifest with hind limb signs in quadrupeds, likely due to gait mechanics and delayed forelimb involvement. These findings underscore important diagnostic implications: clinicians should maintain a high index of suspicion for spinal cord neoplasia when patients present with unexplained hind limb motor deficits, potentially aiding earlier detection.

Previous work has examined spinal cord tumors within single species, such as cats and dogs (e.g. Koestner & Higgins 2007 in cats; Rossmeisl *et al.* 2013 in dogs) [6, 13]. These studies were among the first to collect broad data on immunohistochemistry, clinical features, and tumor distribution for specific domestic species. Moreover, other investigations using animal models have elucidated novel ways to study this disease process (e.g. Caplan *et al.* 2006, describing a rat model of intramedullary spinal tumors) [14]. However, those prior studies each focused on one species. We believe our research is important because we are the first to examine this disease process in a holistic, multi-species manner, summarizing all the available veterinary data across species. This interdisciplinary approach to spinal cord tumors allows human clinicians to gain further insight into the origins and behavior of these tumors. Practical applications can be derived from these comparative data, for instance, developing or refining animal models that mirror human spinal tumors in terms of anatomical location, clinical symptoms, or molecular genetics.

Although our study is the first to review the distribution of intradural spinal tumors across such diverse species, there are some key limitations to acknowledge, including reporting bias, generalizability, and lack of standardization. We also limited the review to terrestrial vertebrates; aquatic taxa (e.g. fish) were outside the scope of this analysis owing to distinct spinal biology and sparse documentation of histologically confirmed intradural spinal tumors. Future work dedicated to aquatic species could evaluate whether the cross-species patterns we observed extend beyond amniotes. While the prevalence and incidence of spinal cord tumors are relatively low in humans, they are virtually unknown in most wild animal species. This gap is driven in part by substantial underdiagnosis: animals cannot communicate subjective symptoms like pain, paresthesia, or early weakness hallmarks, early signs in human spinal tumor cases. In wild populations, the onset of paralysis or neurologic dysfunction often leads rapidly to death by predation, starvation, or exposure, preventing recognition or necropsy of the underlying disease. Thus, only domesticated animals and those in captive environments (e.g. pets, zoo animals, research subjects) with access to veterinary care are likely to receive a diagnosis, which introduces a strong sampling bias toward species that live in proximity to humans. As for generalizability, the disease process of these tumors likely varies between species, but this variation also presents an opportunity, as discussed, to investigate shared vulnerabilities or protective adaptations across species. Lastly, the reporting of these tumors is not standardized. The way clinical presentation, disease course, pathological processing, and histopathological findings are documented often differs from case to case, making direct comparisons difficult, but also demonstrating the need for improved documentation and standardized workups when these tumors are observed in non-human species. Consistent with these constraints, although we assembled species level life history variables, the data were too sparse and heterogeneous to support defensible cross-species correlations (e.g. ‘tumor frequency vs body mass’); we therefore refrained from such exploratory analyses to avoid over interpretation. In our qualitative review, we did not observe a reproducible association between tumor occurrence and either body size or lifespan, and we outline how future, systematically ascertained datasets could enable those analyses. We further recognize that our reliance on published cases introduces potential publication bias, as dramatic or unusual cases are more likely to be reported in the literature. In the future, more systematic approaches, for example, routine necropsy programs in zoological institutions or the integration of wildlife pathology databases, could help capture less conspicuous cases and provide a more balanced understanding of tumor incidence across species. We also acknowledge that by excluding experimental animal model studies, our review may have missed mechanistic insights that laboratory models offer. Incorporating data from well-validated experimental models into comparative analyses (where appropriate) could illuminate shared oncogenic pathways and validate hypotheses generated from spontaneous case data. By addressing these various limitations with targeted strategies, future research can build on our findings with more robust and comprehensive datasets.

**Figure 2:**
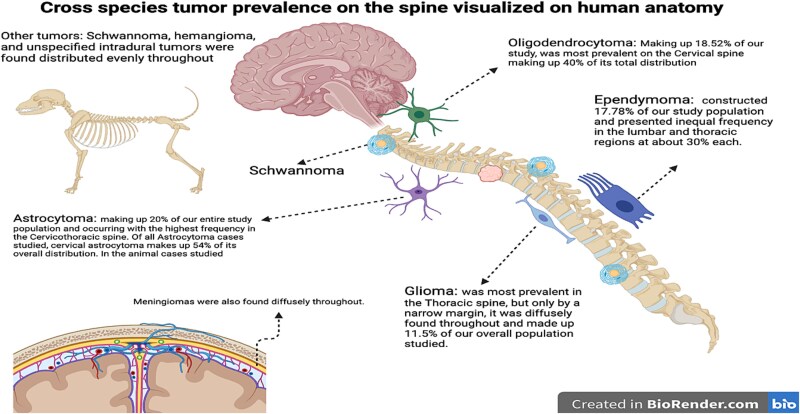
Cross-species tumor distribution mapped onto human spinal anatomy. Astrocytomas were most frequent (20%) and concentrated in the cervical spine (54%). Oligodendrogliomas (18.5%) also favored the cervical region. Ependymomas (17.8%) showed near-equal lumbar and thoracic distribution. Gliomas were diffuse, with a thoracic preference (11.5%). Schwannomas, hemangiomas, meningiomas, and unspecified intradural tumors were evenly distributed.

**Figure 3:**
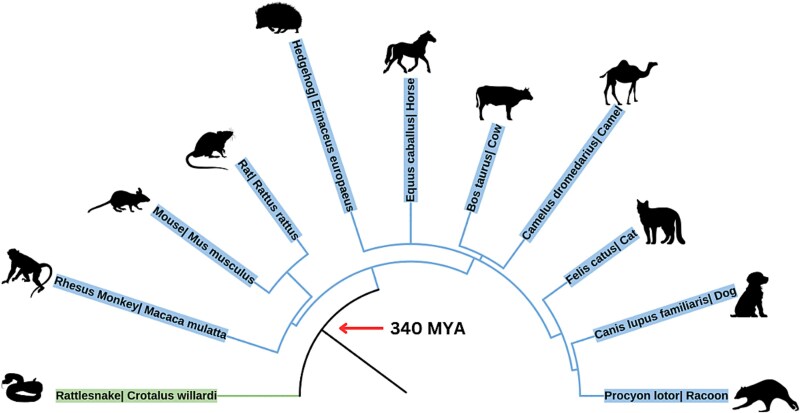
Phylogenetic tree showing distribution of spinal tumor types across 11 species, highlighting clustering of glial tumor vulnerability along evolutionary lines.

As illustrated by our findings and aided by new visualizations, there are clear links between our results and the figures. Figure 2, for instance, maps the cross-species tumor distribution onto human spinal anatomy, revealing that a majority of the glial tumors in animals (especially astrocytomas and oligodendrogliomas) clustered in the cervical spine. This anatomical clustering is important, as it suggests that clinicians should maintain a high index of suspicion for cervical intramedullary tumors when encountering relevant symptoms in both animals and humans. Any comparisons to human distributions are descriptive and hypothesis generating; we did not perform inferential statistics to test cross-species equivalence in tumor type or regional distributions. Figure 3 provides a phylogenetic perspective, showing how various spinal tumor types are distributed across the evolutionary tree. Notably, glial tumors appear in distantly related lineages, reinforcing our hypothesis that fundamental vulnerability to these tumors is deeply conserved. We have created Figs 1–3 specifically for this manuscript using the data from our review (with tools like BioRender and iTOL); all illustrations are original and appropriately licensed.

Looking forward, the findings of this review offer several promising avenues for advancing human spinal tumor research. The consistency of glial-derived tumors across species supports the development of comparative, genetically informed animal models that can be leveraged to investigate conserved oncogenic mechanisms and test novel therapeutic strategies [15]. Mapping the clinical presentation of spinal tumors across species may also help identify symptom patterns or molecular markers useful for earlier diagnosis in humans. Furthermore, our work highlights the need for a more unified, cross-disciplinary research infrastructure that integrates veterinary pathology, evolutionary biology, and translational oncology. Expanding access to large-scale veterinary tumor registries and genomic sequencing efforts could uncover shared genetic drivers and reveal new targets for therapy. Ultimately, by bridging human and non-human data, we can better understand the biology of spinal cord tumors and design more precise, predictive interventions for patients.

## CONCLUSIONS

This study represents the first cross-species synthesis of spontaneous intradural spinal cord tumors in non-human animals, with a particular emphasis on intramedullary glial neoplasms such as astrocytomas, oligodendrogliomas, and ependymomas. We observed consistent patterns in histology, neuroanatomical distribution, and clinical symptoms, including limb paralysis and ataxia that qualitatively resemble presentations reported in human intramedullary tumors; however, these cross-species parallels are descriptive only, as we did not perform inferential testing against human datasets. These findings highlight conserved mechanisms of spinal cord oncogenesis and reinforce the evolutionary relevance of glial tumor vulnerability. Moving forward, there is strong potential to develop genetically informed animal models that reflect these tumor types, enabling more precise investigation into pathogenesis, early diagnostic biomarkers, and therapeutic targets. Expanding tumor registries across veterinary and zoological contexts may also uncover novel insights applicable to early detection and treatment in humans. Ultimately, by narrowing our focus on intradural spinal cord tumors and using comparative data across species, we can strengthen the translational bridge between animal and human spinal oncology.

## Supplementary Material

EVo_Table_S1_eoaf028

Evo_Table_S2_eoaf028
