# Supplementary material for: Evolutionary origins of spinal cord tumors: A cross-species systematic review
Source: Evol Med Public Health. 2025 Oct 14;13(1):365–73. doi: 10.1093/emph/eoaf028 (PMC12640201; doi:10.1093/emph/eoaf028)
Supplement: EVo_Table_S1_eoaf028 [file evo_table_s1_eoaf028.docx]

*Supplementary Table S1. Detailed Neurological Signs (n = 155)*

| **Specific sign** | **n** | **% of all cases** |
| --- | --- | --- |
| Absent deep tendon reflexes | 1 | 0.6% |
| Anal sphincter paralysis | 1 | 0.6% |
| Blindness | 1 | 0.6% |
| Congenital spinal deformation | 4 | 2.6% |
| Difficulty moving the neck | 6 | 3.9% |
| Disordered eating | 1 | 0.6% |
| Forelimb monoparesis | 2 | 1.3% |
| Forelimb ataxia | 2 | 1.3% |
| Forelimb paralysis | 2 | 1.3% |
| Hindlimb monoparesis | 4 | 2.6% |
| Hindlimb ataxia | 38 | 24.5% |
| Hindlimb paralysis | 31 | 20.0% |
| Lethargy | 1 | 0.6% |
| n/a (unspecified) | 26 | 16.8% |
| Priapism | 1 | 0.6% |
| Seizures | 2 | 1.3% |
| Tetraparesis | 21 | 13.5% |
