# Supplementary material for: Evolutionary origins of spinal cord tumors: A cross-species systematic review
Source: Evol Med Public Health. 2025 Oct 14;13(1):365–73. doi: 10.1093/emph/eoaf028 (PMC12640201; doi:10.1093/emph/eoaf028)
Supplement: Evo_Table_S2_eoaf028 [file evo_table_s2_eoaf028.docx]

*Supplementary Table S2. Tumor‐Type Breakdown by Spinal Region*

| \| **Cervical (n = 31)** \| \| \| \| --- \| --- \| --- \| \| **Tumor type** \| **n** \| **% of region** \| \| Astrocytoma \| 9 \| 29.0% \| \| Oligodendroglioma \| 10 \| 32.3% \| \| Ependymoma \| 5 \| 16.1% \| \| Glioblastoma \| 1 \| 3.2% \| \| Intradural, NOS \| 1 \| 3.2% \| \| Lymphoma \| 2 \| 6.5% \| \| Nephroblastoma \| 1 \| 3.2% \| \| Schwannoma \| 2 \| 6.5% \| \| **Cervicothoracic (n = 8)** \| \| \| \| **Tumor type** \| **n** \| **% of region** \| \| Astrocytoma \| 4 \| 50.0% \| \| Glioblastoma \| 2 \| 25.0% \| \| Ependymoma \| 1 \| 12.5% \| \| Hemangioblastoma \| 1 \| 12.5% \| \| **Full spine (n = 18)** \| \| \| \| **Tumor type** \| **n** \| **% of region** \| \| Astrocytoma \| 3 \| 16.7% \| \| Oligodendroglioma \| 3 \| 16.7% \| \| Ependymoma \| 1 \| 5.6% \| \| Glioblastoma \| 2 \| 11.1% \| \| Hemangioblastoma \| 2 \| 11.1% \| \| Meningioma \| 3 \| 16.7% \| \| Adenocarcinoma \| 1 \| 5.6% \| \| Polymorphic cell sarcoma \| 1 \| 5.6% \| \| Schwannoma \| 2 \| 11.1% \| \| **Thoracic (n = 29)** \| \| \| \| **Tumor type** \| **n** \| **% of region** \| \| Ependymoma \| 7 \| 24.1% \| \| Glioblastoma \| 5 \| 17.2% \| \| Astrocytoma \| 3 \| 10.3% \| \| Intradural, NOS \| 5 \| 17.2% \| \| Oligodendroglioma \| 3 \| 10.3% \| \| Schwannoma \| 2 \| 6.9% \| \| Hemangioblastoma \| 2 \| 6.9% \| \| Meningioma \| 1 \| 3.4% \| \| Neuroblastoma \| 1 \| 3.4% \| \| **Thoracolumbar (n = 13)** \| \| \| \| **Tumor type** \| **n** \| **% of region** \| \| Astrocytoma \| 3 \| 23.1% \| \| Ependymoma \| 3 \| 23.1% \| \| Glioblastoma \| 2 \| 15.4% \| \| Oligodendroglioma \| 2 \| 15.4% \| \| Choroid plexus tumor \| 1 \| 7.7% \| \| Intradural, NOS \| 1 \| 7.7% \| \| Nephroblastoma \| 1 \| 7.7% \| \| **Lumbar (n = 28)** \| \| \| \| **Tumor type** \| **n** \| **% of region** \| \| Ependymoma \| 7 \| 25.0% \| \| Astrocytoma \| 5 \| 17.9% \| \| Intradural, NOS \| 6 \| 21.4% \| \| Oligodendroglioma \| 4 \| 14.3% \| \| Polymorphic cell sarcoma \| 2 \| 7.1% \| \| Glioblastoma \| 1 \| 3.6% \| \| Hemangioblastoma \| 1 \| 3.6% \| \| Neuroblastoma \| 2 \| 7.1% \| \| **Lumbosacral (n = 4)** \| \| \| \| **Tumor type** \| **n** \| **% of region** \| \| Oligodendroglioma \| 2 \| 50.0% \| \| Glioblastoma \| 1 \| 25.0% \| \| Neuroblastoma \| 1 \| 25.0% \| \| **Sacral (n = 3)** \| \| \| \| **Tumor type** \| **n** \| **% of region** \| \| Glioblastoma \| 1 \| 50.0% \| \| Oligodendroglioma \| 1 \| 25.0% \| \| Schwannoma \| 1 \| 25.0% \| \| **Spinobulbar (n = 1)** \| \| \| \| **Tumor type** \| **n** \| **% of region** \| \| Schwannoma \| 1 \| 100.0% \| |
| --- | --- | --- | --- | --- | --- | --- | --- | --- | --- | --- | --- | --- | --- | --- | --- | --- | --- | --- | --- | --- | --- | --- | --- | --- | --- | --- | --- | --- | --- | --- | --- | --- | --- | --- | --- | --- | --- | --- | --- | --- | --- | --- | --- | --- | --- | --- | --- | --- | --- | --- | --- | --- | --- | --- | --- | --- | --- | --- | --- | --- | --- | --- | --- | --- | --- | --- | --- | --- | --- | --- | --- | --- | --- | --- | --- | --- | --- | --- | --- | --- | --- | --- | --- | --- | --- | --- | --- | --- | --- | --- | --- | --- | --- | --- | --- | --- | --- | --- | --- | --- | --- | --- | --- | --- | --- | --- | --- | --- | --- | --- | --- | --- | --- | --- | --- | --- | --- | --- | --- | --- | --- | --- | --- | --- | --- | --- | --- | --- | --- | --- | --- | --- | --- | --- | --- | --- | --- | --- | --- | --- | --- | --- | --- | --- | --- | --- | --- | --- | --- | --- | --- | --- | --- | --- | --- | --- | --- | --- | --- | --- | --- | --- | --- | --- | --- | --- | --- | --- | --- | --- | --- | --- | --- | --- | --- | --- | --- | --- | --- | --- | --- | --- | --- | --- | --- | --- | --- | --- | --- | --- | --- | --- | --- | --- | --- | --- | --- | --- | --- | --- | --- | --- | --- | --- | --- | --- | --- | --- | --- | --- |
